# Associations between the Bovine Myostatin Gene and Milk Fatty Acid Composition in New Zealand Holstein-Friesian × Jersey-Cross Cows

**DOI:** 10.3390/ani10091447

**Published:** 2020-08-19

**Authors:** Ishaku L. Haruna, Yunhai Li, Ugonna J. Ekegbu, Hamed Amirpour-Najafabadi, Huitong Zhou, Jon G. H. Hickford

**Affiliations:** Gene-Marker Laboratory, Department of Agricultural Sciences, Faculty of Agriculture and Life Sciences, Lincoln University, Lincoln 7647, New Zealand; Ishaku.Haruna@lincolnuni.ac.nz (I.L.H.); Yunhai.Li@Lincolnuni.ac.nz (Y.L.); Jane.Ekegbu@lincolnuni.ac.nz (U.J.E.); Hamed.Amirpour@lincolnuni.ac.nz (H.A.-N.); zhouh@lincoln.ac.nz (H.Z.)

**Keywords:** myostatin gene, variation, milk, fatty acid, cattle

## Abstract

**Simple Summary:**

The gene that encodes myostatin influences more than one trait, and its expression has been observed in skeletal muscle, as well as the mammary gland. In this study, association analysis revealed that variation in the bovine myostatin gene affects milk fatty acid composition, raising the possibility that this genetic variation may be utilized to increase the amount of unsaturated fatty acid and decrease the amount of saturated fatty acid in milk.

**Abstract:**

The myostatin gene (*MSTN*), which encodes the protein myostatin, is pleiotropic, and its expression has been associated with both increased and decreased adipogenesis and increased skeletal muscle mass in animals. In this study, the polymerase chain reaction, coupled with single strand conformation polymorphism analysis, was utilized to reveal nucleotide sequence variation in bovine *MSTN* in 410 New Zealand (NZ) Holstein-Friesian × Jersey (HF × J)-cross cows. These cows ranged from 3 to 9 years of age and over the time studied, produced an average 22.53 ± 2.18 L of milk per day, with an average milk fat content of 4.94 ± 0.17% and average milk protein content of 4.03 ± 0.10%. Analysis of a 406-bp amplicon from the intron 1 region, revealed five nucleotide sequence variants (*A–E*) that contained seven nucleotide substitutions. Using general linear mixed-effect model analyses the *AD* genotype was associated with reduced C10:0, C12:0, and C12:1 levels when compared to levels in cows with the *AA* genotype. These associations in NZ HF × J cross cows are novel, and they suggest that this variation in bovine *MSTN* could be explored for increasing the amount of milk unsaturated fatty acid and decreasing the amount of saturated fatty acid.

## 1. Introduction

Improving the efficiency of cattle production systems can be achieved by selecting for fast growing animals with increased muscling that have desirable maternal reproductive traits, good milk production, and good mothering ability. To achieve this, it is important to have an understanding of the genes that underpin muscularity and adiposity.

The gene for the protein myostatin (MSTN; gene *MSTN*), which is also called the growth and differentiation factor 8 gene (*GDF8*) has pleiotropic effects. Its expression has been associated with decreased adipogenesis and increased skeletal muscle mass as a result of decreased secretion of leptin [1,2,3].

Sequence variation in *MSTN* has been associated with increases in growth and muscling traits in several species. For example, in cattle it has been connected with having increased numbers of muscle fibers (otherwise known as “double-muscling”) in a number of breeds [4]. Similarly, sequence variation in the first intron has been found to influence growth and carcass traits such as the yield of leg, loin, and total lean meat in NZ Romney sheep [5].

While there is well-documented evidence describing how variation in *MSTN* is associated with growth and muscle traits in beef cattle breeds, there is little information about the effects of *MSTN* nucleotide sequence variation on milk yield and milk fatty acid (FA) composition. There are suggestions that *MSTN* could affect lactation by affecting the production of fatty acid in milk, and primarily through MSTN deficiency being associated with decreased adipogenesis [2,3,6].

The fat of milk from dairy cattle is approximately 70% saturated fatty acid (SFA), 25% monounsaturated fatty acid (MUFA), and 5% polyunsaturated fatty acid (PUFA) [7]. From a human health perspective, an increase in unsaturated fatty acid (UFA) content and a decrease in SFA content could be considered favorable; and equally from a physical perspective (e.g., increased spread-ability of butter), increased relative levels of UFA would be desirable. Previously, it has been identified that the concentrations of different fatty acids in milk are affected most by four parameters: the diet of the cow [8,9], genetic variation between cows within a breed [10,11], breed differences [12,13], and the number of days in milk [14]. In their report, Strucken et al. [14] established that cows in early lactation stage are characterized by negative energy balance as the dietary energy intake is unable to meet the demands of high milk production in approximately the first 60 days of lactation. To offset this balance, an alternative energy source is needed. This leads to the mobilization of body energy stores to balance the deficit between feed intake, and energy expenditure on maintenance and milk production [15]. However, as a consequence of the cow’s body fat being used up for this purpose, other biological pathways are affected, resulting in a change in milk composition. The Holstein-Friesian and the Jersey breeds have one of the most notable differences in terms of the composition of milk fatty acids. Milk from Jersey cows tend to have higher concentrations of some short- and medium-chain length SFA, but lower concentrations of some UFA [16]. These differences could be capitalized upon in order to obtain the preferred fatty acid profile through cross-breeding of these breeds.

In the above context, the objective of this research was to explore the effects of *MSTN* variation on key milk production traits and composition of milk fatty acids in Holstein-Friesian × Jersey (HF × J)-cross (alternatively known as Kiwicross™) dairy cows in Hamilton, New Zealand (NZ). This cross is now the preferred cow for dairy production in NZ and it constitutes more than half of all the dairy cows milked annually. Our working hypothesis is that variation in the gene will affect some milk traits.

## 2. Materials and Methods

### 2.1. The Dairy Cattle Investigated and the Collection of Milk Samples from Them

This research was approved by the Lincoln University Animal Ethics Committee (AEC Approval Number 521). This is a mandated and registered committee that was established, and is regularly audited, under the provisions of the New Zealand Government’s Animal Welfare Act 1999 (http://www.legislation.govt.nz/act/public/1999/0142/latest/DLM49664.html). A total of 410 HF × J-cross dairy cows (alternatively known as Kiwicross™ cows, in Hamilton, New Zealand), of individually variable and unidentified breed ratio, and of 3 to 9 years of age were investigated. The cows were managed in two herds on the Lincoln University Dairy Farm (Canterbury, New Zealand), and all of them were grazed outdoors at all times on pasture (a blend of white clover and perennial ryegrass). All of the cows calved over the months of August-September (spring in the Southern Hemisphere) and they were then milked twice a day for up to 10 months.

Milk samples for gross milk trait analysis were collected once a month from September to February and the daily milk yield in liters was recorded using Tru-test milk meters (Tru-test Ltd., Auckland, New Zealand) and Fourier-transform infra-red spectroscopy (MilkoScan FT 120 Foss, Hillerød, Denmark) was used to analyze milk samples for fat percentage (%) and protein percentage (%). Average daily milk yield, and average protein and fat percentages were calculated over the 6 months of milk collection. The milk samples for fatty acid (FA) analysis were collected at 148 ± 19 days in milk from each cow in a single afternoon milking in mid-January (the middle of summer in the Southern Hemisphere). These were frozen at −20 °C, and then freeze-dried, prior to being individually ground to a fine powder for component analysis.

### 2.2. Gas Chromatography of the Fatty Acids in the Milk Samples

The FAs were methylated and extracted in n-heptane, before being analyzed by gas chromatography (GC) as FA methyl esters (FAMEs). The methylation reactions were performed in 10-mL Kimax tubes. Individual freeze-dried and powdered milk samples (0.17 g) were dissolved in 900 μL of n-heptane (100%, AR grade), before 100 μL of internal standard (5 mg/mL of C21:0 methyl ester in n-heptane) and 4.0 mL of 0.5 M NaOH (in 100% anhydrous methanol) were added.

The tubes were vortexed then incubated in a block heater (Ratek Instruments, Australia) at 50 °C for 15 min. After cooling to room temperature, another 2.0 mL of n-heptane and 2.0 mL of deionized water were added to each tube. After vortexing, the tubes were centrifuged (Megafuge 1.0R, Heraeus, Germany) for 5 min at 1500× *g*. The top layer of n-heptane was transferred into a second Kimax tube and 2.0 mL of n-heptane was added to each of the original tubes. The extraction was repeated and the n-heptane aspirates were then pooled. Anhydrous sodium sulphate (10 mg) was added to the n-heptane extracts, to remove any residual water.

The GC analysis was carried out using a Shimadzu GC-2010 Gas Chromatograph (Shimadzu Corporation, Kyoto, Japan) equipped with a flame ionization detector and an AOC-20i auto sampler. The output was analyzed with GC Solution Software (Shimadzu). For analysis, 1 μL of the n-heptane sample extract was injected into a 100 m GC column (250 μm × 0.25 μm capillary column, CP-Select, Varian) with a 1:60 split ratio. The separation was undertaken with a helium carrier gas and was run for 92 min. The temperature of both the injector and detector were set at 250 °C and the thermal profile of the column consisted of 45 °C for 4 min, followed by 27 min at 175 °C (ramped at 13 °C/min), 35 min at 215 °C (ramped at 4 °C/min), and a final “bake-off” at 250 °C for 5 min (ramped at 25 °C/min). The individual FAMEs were identified by the peak retention time compared to commercially obtained external standards (ME61, ME93, BR3, BR2, ME100, GLC411, and GLC463; Laroden AB, Sweden). Quantification of the individual FAMEs was based on peak area assessment and comparison with the internal and external standards. The threshold for peak area determination on the chromatogram was a 500-unit count, with peaks that were under 500-unit count, being ignored. The calculated minimum component of an individual FAME was therefore 0.01 g per 100 g of total FA.

After their individual measurement, the FAs were arranged into various groups and indices. These groups were, short-chain length FAs (SCFA) = C4:0 + C6:0 + C8:0; medium-chain length FAs (MCFA) = C10:0 + C12:0 + C14:0; long-chain length FAs (LCFA) = C15:0 + C16:0 + C17:0 + C18:0 + C19:0 + C20:0 + C22:0 + C24:0; omega 3 FAs = C18:3 cis-9, 12, 15 + C20:5 cis-5,8, 11, 14, 17 + C22:5 cis-7, 10, 13, 16, 19; omega 6 FAs = C18:2 cis-9, 12 + C18:3 cis-6, 9, 12 + C20:3 cis-8, 11, 14 + C20:4 cis-5, 8, 11, 14; monounsaturated FAs (MUFA) = C10:1 + C12:1 + C14:1 cis-9 + C15:1 + C16:1 cis-9 + C17:1 + C18:1 trans-11 + C18:1 cis-9 + C18:1 cis-(10 to 15) + C20:1 cis-5 + C20:1 cis-9 + C20:1 cis-11 + C22:1 trans-13; polyunsaturated FAs (PUFA) = C18:2 trans-9, 12 + C18:2 cis-9,trans-13 + C18:2 cis-9,trans-12 + C18:2 trans-9,cis-12 + C18:2 cis-9, 12 + C18:3 cis-6, 9, 12 + C18:3 cis-9, 12, 15 + CLA + C20:3 cis-8, 11, 14 + C20:4 cis-5, 8, 11, 14 + C20:5 cis-5, 8, 11, 14, 17 + C22:5 cis-7, 10, 13, 16, 19; and total branched FA = C13:0 *iso* + C13:0 *anteiso* + C15:0 *iso* + C15:0 *anteiso* + C17:0 *iso*.

Unsaturated FA indices were also calculated as follows: C12:1 index (C12:1 divided by the sum of C12:0 and C12:1); C16:1 index (C16:1 cis-9 divided by the sum of C16:0 and C16:1 cis-9); and C18:1 index (C18:1 cis-9 divided by the sum of C18:0 and C18:1 cis-9). The method is as described by Li et al. [17], with the un-adjusted mean levels in the 430 cows being calculated and used subsequently in the statistical analyses.

### 2.3. Blood Sample Collection

Samples of blood were collected from each of the cows studied onto FTA™ cards (Whatman™, Middlesex, UK) by piercing the ear of the animal. This is allowed under a Code of Welfare issued by the NZ Minister of Agriculture, under Section 75 and 76 of the NZ Animal Welfare Act 1999. The samples were allowed to dry in the air and the purification of the DNA from 1.2-mm punches taken from the FTA™ cards was carried out using a two-step procedure described by Zhou et al. [18].

### 2.4. Polymerase Chain Reaction (PCR) Amplification of a Region of the Cattle Myostain Gene

The intron 1 region of *MSTN* was amplified using forward and reverse primers (5′-catggtactattgttgagag-3′ and 5′-aaggcaaatctattccagg-3′ respectively) adapted from the work of Haruna et al. [19]. The 15-µL reactions contained the purified DNA on a 1.2-mm diameter disc of the FTA™ card, and a content of 0.25 µM for each primer, 150 µM for each dNTP (Eppendorf, Hamburg, Germany), 3.0 mM Mg^2+^, 0.5 U of *Taq* DNA polymerase (Qiagen, Hilden, Germany), and 1× the buffer supplied with the DNA polymerase enzyme.

The PCR amplifications were carried out in Bio-Rad S1000 thermal cyclers (Bio-Rad, Hercules, CA, USA) and the thermal cycling parameters included an initial denaturation at 94 °C for 2 min, and then 35 repeated cycles of denaturation at 94 °C for 30 s, primer annealing at 58 °C for 30 s, and primer extension at 72 °C for 30 s. Following this process, a final extension step at 72 °C for 5 min was used.

### 2.5. Single Strand Conformation Polymorphism (SSCP) Analysis

An SSCP technique was employed to detect nucleotide sequence variation in the amplicons obtained from the PCR reactions. A 0.7-µL aliquot of the completed reactions was added to 7 µL of a solution containing 10 mM ethylenediaminetetraacetic acid (EDTA), 0.025% bromophenol blue, 0.025% xylene-cyanol, and 98% formamide. The samples were then placed on a hot plate already set at 95 °C for 5 min for denaturation, followed by immediate cooling on wet ice. They were then loaded onto 14% acrylamide: bisacrylamide (37.5:1) (Bio-Rad) gels that were 16 cm × 18 cm in size. Electrophoresis was undertaken using Protean II xi cells (Bio-Rad) for 19 h at 390 volts and 7 °C room temperature in 0.5 × Tris/Borate/EDTA running buffer.

To detect the SSCP banding patterns, the SSCP gels were stained using the silver-staining method of Byun et al. [20].

### 2.6. Nucleotide Sequencing and Sequence Analysis

Based on the PCR-SSCP patterns observed nucleotide sequence variation could be identified. Cows that appeared to be homozygous with unique banding patterns were subjected to direct sequencing. For cows that appeared to have heterozygous variant patterns, unique bands were excised from the wet gel and incubated in water at 69 °C for 1 h. A 1-µL aliquot of the water product was pipetted into 14-µL of PCR pre-mixture (as used for the original PCR reactions), re-amplified using the same thermal cycle profile, and subsequently sequenced. This approach has been described in more detail by Gong et al. [21]. The sequences were aligned, and other analyses were undertaken using version 5.2.10 of DNAMAN (Lynnon BioSoft, Vaudreuil, QC, Canada).

### 2.7. Statistical Analyses

All statistical analyses were performed using IBM SPSS version 22 (IBM, Armonk, NY, USA), and an alpha level of *p* < 0.05 was set as a threshold.

For genotypes with a frequency greater than 5% (thus having adequate sample size per group), the effect of variation in a cow’s *MSTN* genotype on gross milk production traits, and the component levels of individual and grouped FAs was tested using general linear mixed-effects models (GLMMs) and multiple pair-wise comparisons (least significant difference tests) with Bonferroni corrections. The age of the cow expressed in an integer value of years (i.e., as a categorical variable in a range from 3 to 9 years of age), the number of days in milk for each cow (DIM; expressed as an integer value, but entered into the model as a continuous trait) and herd (to correct for herd-specific effects) were fitted to the models as fixed explanatory factors.

The model was Y_ijkl_ = μ + G_i_ + A_j_ + D_k_ + H_l_ + e_ijkl_ for genotype: where Y_ijkl_ = the observed trait value in the ijklth cow; μ = the mean trait value for a given trait; G_i_ = the fixed effect of ith *MSTN* genotype; A_j_ = effect of age (j = 3–9 years); D_k_ = effect of the number of days the cow has produced milk (DIM: k = 94–186 days); H_l_ = the fixed effect of lth farm (l = 1 or 2); and e_ijkl_ = random error.

The effect of sire of cow could not be included in the GLMMs, because some semen straws (sire genetics) used in NZ dairy cattle artificial insemination-based breeding approaches contain mixed-sire semen purchased from commercial semen producers. In these cases, it is impossible to ascertain individual sire identity. However, since the straws were mixed-semen straws and because different sires are used for different inseminations, in different years, it is unlikely that sire was a strongly confounding effect. Cow age and herd might also be confounded with sire, but this cannot be confirmed.

## 3. Results

### 3.1. Milk Production

Over the time the cows were studied, they produced an average of 22.53 ± 2.18 L of milk per day, with an average milk fat content of 4.94 ± 0.17% and average milk protein content of 4.03 ± 0.10%.

### 3.2. Identification of Nucleotide Sequence Variation in Bovine MSTN

A 406 bp fragment of the intron 1 region of bovine *MSTN* was amplified and analyzed using the polymerase chain reaction coupled with single strand conformation polymorphism (PCR-SSCP) analyses.

**Figure 1:**
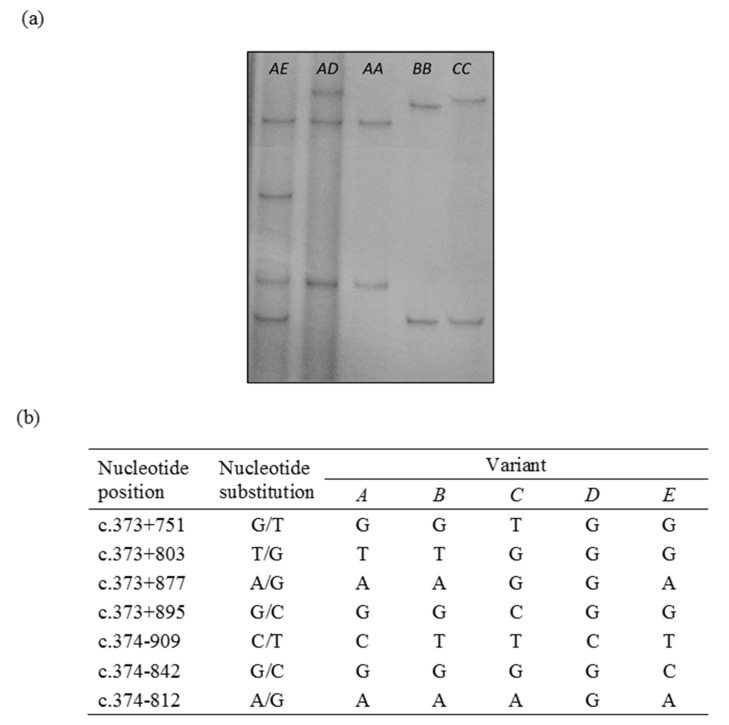
Variation in intron 1 of bovine *MSTN* revealed using PCR-SSCP analysis (**a**) and verified with nucleotide sequencing (**b**). Five PCR-SSCP patterns, representing the five distinct sequence variants (*A–E*, in both homozygous and heterozygous genotypes) are shown.

The PCR-SSCP analyses coupled with DNA sequencing revealed five banding patterns (*A-E*) in the region of intron 1 investigated (Figure 1). A total of seven single-nucleotide substitutions (c.373+751G/T, c.373+803T/G, c.373+877A/G, c.373+895G/C, c.374−909C/T, c.374−842G/C, c.374−812A/G) were identified, all of which have been previously reported [19].

### 3.3. MSTN Genotype Models

The genotypes *AA* (*n* = 149), *AB* (*n* = 87), *AC* (*n* = 48) and *AD* (*n* = 63) occurred at a frequency over 5% and were analyzed in this model. The other genotypes *AE* (*n* = 16), *BB* (*n* = 6), *BC* (*n* = 5), *BD* (*n* = 15), *CC* (*n* = 5), *CD* (*n* = 7), CE (*n* = 1) and *DD* (*n* = 3) had frequencies of less than 5% each, and were not included in the model.

In the genotype models (Table 1), genotype *AD* was associated with a lower C12:0 and C12:1 levels than genotype *AA*.

## 4. Discussion

This is the first study demonstrating association of *MSTN* sequence variants with the component levels of two milk FAs in NZ HF × J cross cattle.

All seven of the nucleotide substitutions identified in this study have been previously reported in a study of NZ cattle breeds, which included; Hereford, Angus, Shorthorn, Charolais, Red Poll, South Devon, Simmental, Murray Grey, HF × J cross cattle, and some composite breeds [19]. The nucleotide substitutions c.373+751G/T, c.373+803T/G, c.373+877A/G, c.373+895G/C, and c.374−909C/T were identified in all the ten aforementioned breeds, while c.374−842G/C was found in all but four breeds (Red Poll, Shorthorn, Simmental, and Composites breeds) and the c.374−812A/G was only found in Shorthorn and HF × J cross breeds.

In a previous study, Smith et al. [22] identified a mutant “*mh*” allele of bovine *MSTN* with an 11-bp deletion in the exon 3 region. This was described in British South Devon cattle, and it was the same allele first identified in the Belgian Blue breed, causing the “double-muscled” phenotype. Also, in a later investigation of *MSTN* in 146 British South Devon cattle, Wiener et al. [23] showed that this same mutant “*mh*” allele, reduced the levels of total SFA and total MUFA in the muscle (*p* < 0.05). Their report also revealed an increase in the ratio of PUFA: SFA in total lipid to be greater in *mh/mh* individuals than in the other two genotype (*mh*/+ and +/+) classes (*p* < 0.001). This suggested that the “*mh*” allele is associated with reduced fat levels, particularly with the levels of SFA and MUFA. The increase in the ratio of PUFA to SFA would be expected, especially when the concentration of muscle fat decreases.

In a similar report on associations between the “*mh*” allele carrying the 11-bp deletion with intramuscular fatty acid composition in *MSTN* of Belgian Blue young bulls, Raes et al. [24] revealed that animals with the +/+ (normal) genotype showed a higher relative amount of the SFA; C14:0 and C16:0 and a higher relative amount of all MUFA C16:1; C17:1 and C18:1, but the relative proportion of PUFA in total fatty acids increased with increasing mutant “*mh*” alleles. In the current study, the genotype model revealed that cows carrying the *AA* genotype showed an increase in the amount of two medium-chain length SFAs (C12:0 and C12:1) relative to *AD* cows. While it may be difficult to conclude that the findings in the current study are similar to the work of Weiner et al. [23] and Raes et al. [24], especially because it investigated the intronic region of *MSTN* in non-doubled muscled cattle breed, it is important to note that variations in the intronic regions are equally capable of influencing gene expression and/or altering the functionality of a gene [25]. In a previous investigation, He et al. [25] transformed C2C12 cell-lines with a transgene construct that contained bovine *MSTN* promoter (pMD-MSTNPro) and a second construct that contained the first intron of bovine *MSTN* (pMD-Intron1). They observed an increase in the mean fluorescence intensity of green fluorescent protein (GFP) gene and the percentage of fluorescence positive cells, and concluded from this that the presence of intron 1 of bovine *MSTN* increased the expression of GFP in the transformed cells.

The findings in this study are in part in agreement with the findings of Buske et al. [26]. That study revealed that one copy of the so-called “wild-type + allele” of *MSTN* was associated with higher milk, protein, and fat yields in dual purpose Belgian Blue (DP-BB) cows, whereas a single copy of the mutant “*mh*” allele (associated with double muscling) was associated with a decrease in the SFA content of milk. Even though the current study did not involve the *MSTN* “*mh*” allele, it has perhaps suggested that antagonistic effects may exist between milk and meat production traits in the context of *MSTN*.

Since the *MSTN* variations in the current study occurred in a non-expressed region of the gene, it is perhaps less obvious how they might affect the structure or function of the MSTN protein. They might however influence the rate of transcription and/or translation of the gene, as was described by Liu et al. [27] in an investigation that involved both transgenic mice and in vitro studies. In that study, intron sequences in the transgene that encoded rat growth hormone were observed to stimulate transcription by promoting assembly of an ordered nucleosome array in the vicinity of the promoter. Additionally, while the observed intron variation does not yield any amino acid change, several studies have shown that synonymous nucleotide substitutions can affect the phenotypic characteristics of the protein product by altering mRNA structure, protein stability, the electrical charge of the resulting polypeptide, and codon usage during mRNA translation [28,29].

Previous reports on the differences in the composition of milk FAs between Holstein-Friesian and the Jersey breeds suggest that milk from Jersey cows tends to have higher concentrations of some short- and medium-chain length SFA, but lower concentrations of some UFA [16]. It is therefore interesting to note that the current investigation of NZ HF × J cross cows, revealed that variant *D* was associated with a decrease in the amount of two medium-chain length SFAs in milk. This discovery could be of benefit in terms of its potential applicability in cross-breeding and gene-marker development, particularly in selection for decreased SFA in milk.

## 5. Conclusions

The findings suggest that variation in *MSTN* affects two milk FA traits and this may be of value in breeding dairy cattle. Notably, variant *D* of *MSTN* is associated with a decrease in two medium-chain length SFA levels. Cows with the *AD* genotype might therefore produce a “preferred” FA profile in milk. However, because there were insufficient cattle with the homozygous genotypes *BB*, *CC*, and *DD*, or the heterozygous genotypes (*BC*, *BD*, *CE*, and *CD*) in the samples investigated, further investigation involving much larger sample sizes across different farms and breeds of cattle is needed to validate this claim.

## Figures and Tables

**Table 1:** Associations between milk fatty acid methyl ester levels and myostatin genotypes in NZ HF × J cross cows (*n* = 347).

Individual/Grouped Fatty Acids ^1^	Mean ± Standard Error ^2^ (g/100 g Milk Fatty Acids)				*p* ^3^
	*AA* (*n* = 149)	*AB* (*n* = 87)	*AC* (*n* = 48)	*AD* (*n* = 63)	
C4:0	1.264 ± 0.012	1.252 ± 0.015	1.252 ± 0.020	1.262 ± 0.017	0.871
C6:0	1.566 ± 0.010	1.548 ± 0.013	1.549 ± 0.017	1.547 ± 0.015	0.458
C8:0	1.198 ± 0.009	1.179 ± 0.011	1.188 ± 0.014	1.169 ± 0.012	*0.150*
C10:0	3.299 ± 0.035	3.244 ± 0.043	3.293 ± 0.056	3.164 ± 0.050	*0.097*
C10:1	0.294 ± 0.004	0.282 ± 0.005	0.283 ± 0.007	0.284 ± 0.006	*0.108*
C11:0	0.062 ± 0.002	0.058 ± 0.002	0.056 ± 0.003	0.057 ± 0.002	*0.124*
C12:0	4.029 ± 0.046 ^a^	3.939 ± 0.057 ^ab^	4.015 ± 0.075 ^ab^	3.824 ± 0.067 ^b^	**0.046**
C13:0 *iso*	0.084 ± 0.002	0.079 ± 0.002	0.080 ± 0.002	0.078 ± 0.002	*0.076*
C12:1	0.096 ± 0.002 ^a^	0.091 ± 0.002 ^ab^	0.093 ± 0.003 ^ab^	0.088 ± 0.003 ^b^	**0.019**
C13:0 *anteiso*	0.038 ± 0.000	0.038 ± 0.001	0.038 ± 0.001	0.039 ± 0.001	0.450
C13:0	0.123 ± 0.002	0.119 ± 0.003	0.117 ± 0.004	0.118 ± 0.004	0.364
C14:0	12.513 ± 0.079	12.478 ± 0.098	12.690 ± 0.128	12.365 ± 0.114	0.266
C14:1 *cis*-9	1.003 ± 0.020	0.959 ± 0.025	0.967 ± 0.033	0.966 ± 0.029	0.390
C15:0 *iso*	0.296 ± 0.002	0.290 ± 0.003	0.299 ± 0.004	0.290 ± 0.004	*0.148*
C15:0 *anteiso*	0.639 ± 0.009	0.633 ± 0.011	0.647 ± 0.014	0.646 ± 0.012	0.814
C15:0	1.485 ± 0.016	1.473 ± 0.019	1.474 ± 0.026	1.471 ± 0.023	0.922
C15:1	0.283 ± 0.003	0.281 ± 0.004	0.287 ± 0.005	0.286 ± 0.004	0.714
C16:0	37.396 ± 0.288	37.624 ± 0.354	36.999 ± 0.465	37.558 ± 0.412	0.711
C16:1 *cis*-9	1.266 ± 0.023	1.245 ± 0.028	1.273 ± 0.037	1.301 ± 0.033	0.576
C17:0 *iso*	0.555 ± 0.006	0.553 ± 0.008	0.556 ± 0.010	0.553 ± 0.009	0.987
C17:0	0.690 ± 0.006	0.698 ± 0.008	0.693 ± 0.010	0.690 ± 0.009	0.816
C17:1	0.195 ± 0.002	0.194 ± 0.003	0.198 ± 0.004	0.199 ± 0.003	0.639
C18:0	8.547 ± 0.119	8.652 ± 0.146	8.652 ± 0.192	8.607 ± 0.170	0.918
C18:1 *trans*-11	2.756 ± 0.068	2.782 ± 0.083	2.801 ± 0.110	2.794 ± 0.097	0.974
C18:1 *cis*-9	12.833 ± 0.142	12.891 ± 0.175	12.891 ± 0.230	13.146 ± 0.204	0.595
C18:2 *trans*-9,12	0.377 ± 0.006	0.370 ± 0.007	0.391 ± 0.009	0.381 ± 0.008	0.230
C18:2 *cis*-9, *trans*-13	0.288 ± 0.003	0.281 ± 0.004	0.291 ± 0.005	0.292 ± 0.005	0.225
C18:2 *cis*-9, *trans*-12	0.067 ± 0.002	0.064 ± 0.002	0.065 ± 0.003	0.067 ± 0.002	0.670
C18:2 *trans*-9, *cis*-12	0.475 ± 0.011	0.483 ± 0.014	0.492 ± 0.018	0.473 ± 0.016	0.771
C18:2 *cis*-9,12	0.693 ± 0.007	0.677 ± 0.009	0.697 ± 0.012	0.699 ± 0.011	0.279
C19:0	0.140 ± 0.003	0.140 ± 0.003	0.143 ± 0.004	0.142 ± 0.004	0.890
C18:3 *cis*-6,9,12	0.075 ± 0.001	0.073 ± 0.001	0.074 ± 0.002	0.073 ± 0.001	0.511
C18:3 *cis*-9,12,15	0.805 ± 0.010	0.776 ± 0.013	0.818 ± 0.017	0.800 ± 0.015	*0.144*
CLA *cis*-9, *trans*-11	1.012 ± 0.028	1.001 ± 0.034	1.031 ± 0.045	1.029 ± 0.040	0.920
C20:0	0.126 ± 0.002	0.126 ± 0.002	0.130 ± 0.003	0.127 ± 0.002	0.530
C20:1 *cis*-5	0.060 ± 0.001	0.060 ± 0.002	0.062 ± 0.002	0.060 ± 0.002	0.891
C20:1 *cis*-9	0.151 ± 0.002	0.152 ± 0.003	0.149 ± 0.004	0.151 ± 0.003	0.890
C20:1 *cis*-11	0.077 ± 0.001	0.075 ± 0.002	0.076 ± 0.002	0.073 ± 0.002	0.093
C20:3 *cis*-8,11,14	0.030 ± 0.001	0.031 ± 0.001	0.032 ± 0.001	0.029 ± 0.001	0.137
C20:4 *cis*-5,8,11,14	0.035 ± 0.001	0.035 ± 0.001	0.034 ± 0.001	0.034 ± 0.001	0.927
C22:0	0.064 ± 0.001	0.067 ± 0.002	0.066 ± 0.002	0.066 ± 0.002	0.519
C22:1 *trans*-13	0.066 ± 0.001	0.068 ± 0.002	0.070 ± 0.002	0.067 ± 0.002	*0.190*
C20:5 *cis*-5,8,11,14,17	0.088 ± 0.00	0.089 ± 0.001	0.089 ± 0.002	0.088 ± 0.002	0.936
C24:0	0.044 ± 0.001	0.046 ± 0.001	0.044 ± 0.001	0.045 ± 0.001	0.294
C22:5 *cis*-7,10,13,16,19	0.123 ± 0.002	0.122 ± 0.003	0.123 ± 0.004	0.123 ± 0.003	0.996
SCFA	4.030 ± 0.027	3.979 ± 0.033	3.989 ± 0.043	3.977 ± 0.038	0.451
MCFA	19.841 ± 0.148	19.661 ± 0.182	19.997 ± 0.239	19.353 ± 0.212	*0.127*
LCFA	48.492 ± 0.252	48.826 ± 0.310	48.201 ± 0.407	48.706 ± 0.360	0.587
Total C18:1	16.300 ± 0.168	16.383 ± 0.206	16.400 ± 0.271	16.654 ± 0.240	0.634
Total C18:2	2.911 ± 0.041	2.876 ± 0.051	2.969 ± 0.067	2.940 ± 0.059	0.664
Total C18:3	0.880 ± 0.011	0.849 ± 0.013	0.893 ± 0.017	0.873 ± 0.015	*0.123*
Omega 3	1.017 ± 0.011	0.987 ± 0.014	1.030 ± 0.018	1.011 ± 0.016	*0.173*
Omega 6	0.832 ± 0.008	0.815 ± 0.010	0.838 ± 0.013	0.836 ± 0.011	0.317
MUFA	19.792 ± 0.174	19.790 ± 0.214	19.858 ± 0.281	20.128 ± 0.249	0.657
PUFA	4.067 ± 0.044	4.002 ± 0.054	4.139 ± 0.071	4.088 ± 0.063	0.410
Branched FA	1.611 ± 0.014	1.593 ± 0.017	1.620 ± 0.022	1.605 ± 0.020	0.735
Total UFA	23.859 ± 0.206	23.792 ± 0.254	23.997 ± 0.334	24.216 ± 0.295	0.663
Total SFA	72.548 ± 0.225	72.643 ± 0.277	72.362 ± 0.364	72.210 ± 0.323	0.701
Unsaturated index	24.756 ± 0.218	24.678 ± 0.269	24.911 ± 0.353	25.119 ± 0.313	0.677
C10:1 index	8.252 ± 0.136	8.032 ± 0.167	7.972 ± 0.219	8.321 ± 0.194	0.410
C12:1 index	2.328 ± 0.034	2.239 ± 0.042	2.266 ± 0.055	2.256 ± 0.048	0.243
C14:1 index	7.424 ± 0.148	7.133 ± 0.183	7.087 ± 0.240	7.256 ± 0.213	0.427
C16:1 index	3.281 ± 0.052	3.200 ± 0.063	3.319 ± 0.083	3.338 ± 0.074	0.435
C18:1 index	65.594 ± 0.331	65.409 ± 0.407	65.514 ± 0.535	65.913 ± 0.474	0.855
CLA index	26.626 ± 0.269	26.339 ± 0.332	26.725 ± 0.436	26.868 ± 0.386	0.716

^1^ SCFA- short-chain length fatty acid; MCFA—medium-chain length fatty acid; LCFA—long-chain length fatty acid; MUFA—monounsaturated fatty acid; PUFA—polyunsaturated fatty acid; UFA—unsaturated fatty acid; SFA—saturated fatty acid. ^2^ Predicted means and standard error of those means derived from general linear mixed-effects models (GLMM). Myostatin genotype (categorical variable), cow age (categorical), herd (categorical), and days in milk (continuous) were fitted to the model as fixed effects. Means within a row that do not share a superscript letter are separated at *p* < 0.05. ^3^ 0.05 < *p* < 0.2 in italics, while *p* < 0.05 in bold.

## Supplementary Materials

Supplementary File 1
