# Supplementary material for: Associations between the Bovine Myostatin Gene and Milk Fatty Acid Composition in New Zealand Holstein-Friesian × Jersey-Cross Cows
Source: Animals (Basel). 2020 Aug 19;10(9):1447. doi: 10.3390/ani10091447 (PMC7552700; doi:10.3390/ani10091447)
Supplement: Supplementary File 1 [file animals-10-01447-s001.pdf]

Supplementary Table S1. Associations between *MSTN* variant presence/absence and gross milk production traits

Milk trait	Variant	Mean ± SE[1]				P^2
		Absent	n	Present	n	
Daily milk yield (liters/day)	*A*	23.7 ± 0.58	53	23.0 ± 0.46	377	0.151
	B	22.9 ± 0.46	302	23.6 ± 0.49	128	*0.077*
	C	23.1 ± 0.46	357	23.1 ± 0.56	73	0.984
	D	23.1 ± 0.45	337	23.1 ± 0.54	93	0.816
Fat (%)	*A*	4.8 ± 0.09	53	5.1 ± 0.07	377	*0.064*
	B	4.9 ± 0.07	302	4.9 ± 0.08	128	0.892
	C	5.0 ± 0.07	357	4.9 ± 0.09	73	*0.068*
	D	5.0 ± 0.07	337	4.9 ± 0.08	93	0.368
Protein (%)	*A*	4.0 ± 0.05	53	4.0 ± 0.04	377	*0.079*
	B	4.0 ± 0.04	302	4.0 ± 0.04	128	0.154
	C	4.0 ± 0.04	357	4.0 ± 0.05	73	*0.084*
	D	4.0 ± 0.04	337	4.0 ± 0.04	93	0.244

[1]The predicted means and standard error of those means were derived from the GLMMs. Cow age (age of the cow in years), herd and days in milk (DIM; being the integer number of days from the commencement of lactation for each individual cow), were fitted to the models as fixed effects.

[2]$P < 0.05$ in bold; $0.05 \leq P < 0.10$ in italics

Supplementary Table S2. Associations between *MSTN* variant presence/absence and individual milk fatty acid component levels

Fatty acid (FA)	Variant	Mean FA level ± SE[1] (g/100 g milk FA)				*P*[2]
		Absent	n	Present	n	
C4:0	A	1.28 ± 0.022	53	1.28 ± 0.017	377	0.898
	B	1.28 ± 0.017	302	1.27 ± 0.018	128	0.475
	C	1.28 ± 0.017	357	1.28 ± 0.021	73	0.999
	D	1.28 ± 0.017	337	1.29 ± 0.020	93	0.441
C6:0	A	1.55 ± 0.019	53	1.57 ± 0.014	377	0.479
	B	1.57 ± 0.015	302	1.56 ± 0.015	128	0.396
	C	1.56 ± 0.014	357	1.56 ± 0.018	73	0.722
	D	1.57 ± 0.014	337	1.55 ± 0.017	93	0.294
C8:0	*A*	1.18 ± 0.016	53	1.19 ± 0.013	377	0.343
	B	1.12 ± 0.013	302	1.18 ± 0.014	128	0.164
	C	1.19 ± 0.013	357	1.18 ± 0.015	73	0.917
	D	**1.19 ± 0.012**	**337**	**1.17 ± 0.015**	**93**	**0.021**
C10:0	*A*	3.23 ± 0.064	53	3.25 ± 0.050	377	0.605
	B	3.26 ± 0.051	302	3.22 ± 0.054	128	0.320
	C	3.24 ± 0.050	357	3.26 ± 0.061	73	0.807
	D	**3.28 ± 0.049**	**337**	**3.16 ± 0.058**	**93**	**0.009**
C10:1	*A*	*0.27 ± 0.008*	*53*	*0.29 ± 0.006*	*377*	*0.081*
	B	*0.29 ± 0.006*	*302*	*0.28 ± 0.006*	*128*	*0.066*
	C	0.28 ± 0.006	357	0.28 ± 0.007	73	0.439
	D	0.29 ± 0.006	337	0.28 ± 0.007	93	0.187
C11:0	*A*	0.06 ± 0.003	53	0.06 ± 0.002	377	0.258
	B	0.06 ± 0.002	302	0.06 ± 0.003	128	0.129
	C	*0.06 ± 0.002*	*357*	*0.05 ± 0.003*	*73*	*0.082*
	D	0.06 ± 0.002	337	0.06 ± 0.003	93	0.211
C12:0	*A*	3.90 ± 0.085	53	3.95 ± 0.067	377	0.479
	B	3.96 ± 0.068	302	3.90 ± 0.073	128	0.233
	C	3.94 ± 0.067	357	3.95 ± 0.082	73	0.879
	D	**3.98 ± 0.066**	**337**	**3.82 ± 0.078**	**93**	**0.006**
C12:1	*A*	0.09 ± 0.003	53	0.09 ± 0.002	377	0.184
	B	*0.09 ± 0.003*	*302*	*0.09 ± 0.003*	*128*	*0.050*
	C	0.09 ± 0.002	357	0.09 ± 0.003	73	0.845
	D	**0.09 ± 0.002**	**337**	**0.09 ± 0.003**	**93**	**0.022**
C13:0	*A*	0.11 ± 0.005	53	0.19 ± 0.004	377	0.245
	B	0.12 ± 0.004	302	0.11 ± 0.004	128	0.172
	C	0.12 ± 0.004	357	0.11 ± 0.004	73	0.188
	D	0.12 ± 0.004	337	0.11 ± 0.004	93	0.198

C13:0 *iso*	*A*	0.08 ±0.003	53	0.08 ±0.002	377	0.104
	B	*0.08 ±0.002*	*302*	*0.08 ±0.002*	*128*	*0.097*
	C	0.08 ±0.002	357	0.08 ±0.003	73	0.404
	D	*0.08 ±0.002*	*337*	*0.08 ±0.003*	*93*	*0.090*
C13:0 *anteiso*	*A*	0.04 ±0.001	53	0.04 ±0.001	377	0.763
	B	0.04 ±0.001	302	0.04 ±0.001	128	0.201
	C	0.04 ±0.001	357	0.04 ±0.001	73	0.714
	D	0.04 ±0.001	337	0.04 ±0.001	93	0.623
C14:0	*A*	12.52 ±0.148	53	12.48 ±0.117	377	0.795
	B	12.51 ±0.118	302	12.46 ±0.126	128	0.652
	C	12.45 ±0.116	357	12.61 ±0.141	73	0.170
	D	12.53 ±0.115	337	12.37 ±0.136	93	0.123
C14:1 cis-9	*A*	0.94 ±0.037	53	0.98 ±0.029	377	0.263
	B	0.98 ±0.030	302	0.95 ±0.032	128	0.227
	C	0.97 ±0.029	357	0.96 ±0.035	73	0.590
	D	0.97 ±0.029	337	0.95 ±0.034	93	0.502
C15:0	*A*	1.45 ±0.029	53	1.47 ±0.023	377	0.275
	B	1.47 ±0.023	302	1.46 ±0.024	128	0.438
	C	1.47 ±0.022	357	1.47 ±0.028	73	0.882
	D	1.47 ±0.022	337	1.45 ±0.027	93	0.303
C15:1	*A*	0.28 ±0.005	53	0.28 ±0.004	377	0.346
	B	0.28 ±0.004	302	0.28 ±0.005	128	0.282
	C	0.28 ±0.004	357	0.28 ±0.005	73	0.300
	D	0.28 ±0.004	337	0.28 ±0.005	93	0.650
C15:0 *iso*	*A*	0.29 ±0.005	53	0.29 ±0.004	377	0.333
	B	**0.29 ±0.004**	**302**	**0.29 ±0.004**	**128**	**0.031**
	C	0.29 ±0.004	357	0.29 ±0.004	73	0.148
	D	*0.29 ±0.004*	*337*	*0.29 ±0.004*	*93*	*0.087*
C15:0 *anteiso*	*A*	0.62 ±0.016	53	0.63 ±0.013	377	0.537
	B	0.63 ±0.013	302	0.62 ±0.014	128	0.273
	C	0.62 ±0.013	357	0.63 ±0.015	73	0.361
	D	0.63 ±0.013	337	0.62 ±0.015	93	0.374
C16:0	*A*	36.94 ±0.518	53	37.18 ±0.408	377	0.593
	B	37.01 ±0.412	302	37.29 ±0.441	128	0.388
	C	37.26 ±0.505	357	36.69 ±0.493	73	0.254
	D	37.04 ±0.404	337	37.37 ±0.476	93	0.350
C16:1 *cis*-9	*A*	1.36 ±0.043	53	1.35 ±0.034	377	0.894
	B	1.36 ±0.034	302	1.34 ±0.037	128	0.506
	C	1.36 ±0.034	357	1.35 ±0.041	73	0.853
	D	**1.33 ±0.034**	**337**	**1.40 ±0.040**	**93**	**0.031**
C17:0	*A*	0.70 ±0.012	53	0.70 ±0.683	377	0.920

	B	0.70 ±0.009	302	0.71 ±0.010	128	0.264
	C	0.70 ±0.009	357	0.70 ±0.011	73	0.736
	D	0.70 ±0.009	337	0.70 ±0.011	93	0.339
C17:1	*A*	0.21 ±0.004	53	0.21 ±0.003	377	0.327
	B	0.21 ±0.003	302	0.21 ±0.004	128	0.674
	C	0.21 ±0.003	357	0.21 ±0.004	73	0.281
	D	0.21 ±0.003	337	0.21 ±0.004	93	0.300
C17:0 *iso*	*A*	0.56 ±0.012	53	0.56 ±0.009	377	0.719
	B	0.56 ±0.009	302	0.56 ±0.010	128	0.788
	C	0.56 ±0.009	357	0.57 ±0.011	73	0.253
	D	0.56 ±0.009	337	0.56 ±0.011	93	0.555
C18:0	*A*	8.82 ±0.220	53	8.58 ±0.173	377	0.298
	B	8.60 ±0.175	302	8.71 ±0.187	128	0.429
	C	8.61 ±0.173	357	8.73 ±0.210	73	0.486
	D	8.64 ±0.172	337	8.65 ±0.203	93	0.911
C18:1 *cis*-9	*A*	13.76 ±0.263	53	13.45 ±0.207	377	0.167
	B	13.51 ±0.210	302	13.54 ±0.224	128	0.850
	C	13.48 ±0.206	357	13.64 ±0.251	73	0.445
	D	13.45 ±0.205	337	13.73 ±0.242	93	0.124
C18:1 *trans*-11	*A*	2.52 ±0.125	53	2.64 ±0.099	377	0.277
	B	2.62 ±0.100	302	2.59 ±0.107	128	0.695
	C	2.61 ±0.098	357	2.60 ±0.120	73	0.905
	D	2.63 ±0.098	337	2.54 ±0.115	93	0.302
C18:2 *cis*-9,12	*A*	0.71 ±0.014	53	0.69 ±0.011	377	0.199
	B	*0.70 ±0.011*	*302*	*0.69 ±0.012*	*128*	*0.098*
	C	0.69 ±0.011	357	0.70 ±0.014	73	0.315
	D	0.69 ±0.011	337	0.70 ±0.013	93	0.186
C18:2 *trans*-9,12	*A*	0.35 ±0.010	53	0.35 ±0.008	377	0.330
	B	*0.36 ±0.008*	*302*	*0.34 ±0.009*	*128*	*0.056*
	C	*0.35 ±0.008*	*357*	*0.36 ±0.010*	*73*	*0.090*
	D	0.35 ±0.008	337	0.35 ±0.009	93	0.791
C18:2 cis-9, *trans*-12	*A*	0.07 ±0.003	53	0.07 ±0.002	377	0.786
	B	0.07 ±0.002	302	0.07 ±0.003	128	0.373
	C	0.07 ±0.002	357	0.07 ±0.003	73	0.973
	D	0.07 ±0.002	337	0.07 ±0.003	93	0.312
C18:2 *trans*-9, *cis*-12	*A*	0.44 ±0.021	53	0.45 ±0.016	377	0.408
	B	0.45 ±0.016	302	0.45 ±0.018	128	0.882
	C	0.45 ±0.016	357	0.46 ±0.020	73	0.540
	D	0.45 ±0.016	337	0.44 ±0.019	93	0.203
C18:2 *cis*-9, *trans*-13	*A*	0.29 ±0.006	53	0.29 ±0.005	377	0.306
	B	*0.29 ±0.005*	*302*	*0.28 ±0.005*	*128*	*0.062*

	C	*0.28 ±0.005*	*357*	*0.29 ±0.006*	*73*	*0.064*
	D	0.29 ±0.005	337	0.29 ±0.006	93	0.167
C18:3 *cis*-6,9,12	*A*	0.08 ±0.002	53	0.07 ±0.002	377	0.331
	B	0.07 ±0.002	302	0.07 ±0.002	128	0.462
	C	0.07 ±0.002	357	0.08 ±0.002	73	0.514
	D	0.07 ±0.002	337	0.07 ±0.002	93	0.635
C18:3 *cis*-9,12,15	*A*	0.80 ±0.020	53	0.78 ±0.015	377	0.203
	B	*0.79 ±0.016*	*302*	*0.77 ±0.017*	*128*	*0.061*
	C	0.78 ±0.015	357	0.80 ±0.019	73	0.141
	D	0.78 ±0.015	337	0.79 ±0.018	93	0.425
C20:0	*A*	0.13 ±0.003	53	0.13 ±0.002	377	0.968
	B	0.13 ±0.002	302	0.12 ±0.003	128	0.851
	C	0.12 ±0.002	357	0.13 ±0.003	73	0.275
	D	0.13 ±0.002	337	0.12 ±0.003	93	0.626
C20:1 *cis*-5	*A*	0.06 ±0.003	53	0.06 ±0.002	377	0.597
	B	0.06 ±0.002	302	0.06 ±0.002	128	0.739
	C	0.06 ±0.002	357	0.06 ±0.002	73	0.608
	D	0.06 ±0.002	337	0.06 ±0.002	93	0.647
C20:1 *cis*-9	*A*	0.15 ±0.004	53	0.15 ±0.003	377	0.151
	B	0.15 ±0.003	302	0.15 ±0.004	128	0.798
	C	0.15 ±0.003	357	0.15 ±0.004	73	0.145
	D	0.15 ±0.003	337	0.15 ±0.004	93	0.575
C20:1 *cis*-11	*A*	0.08 ±0.002	53	0.08 ±0.002	377	0.687
	B	0.08 ±0.002	302	0.08 ±0.002	128	0.354
	C	0.08 ±0.002	357	0.08 ±0.002	73	0.823
	D	0.08 ±0.002	337	0.08 ±0.002	93	0.504
C20:3 *cis*-8,11,14	*A*	0.03 ±0.001	53	0.03 ±0.001	377	0.412
	B	0.03 ±0.001	302	0.03 ±0.001	128	0.242
	C	**0.03 ±0.001**	**357**	**0.03 ±0.001**	**73**	**0.017**
	D	0.03 ±0.001	337	0.03 ±0.001	93	0.478
C20:4 *cis*-5,8,11,14	*A*	0.04 ±0.001	53	0.03 ±0.001	377	0.275
	B	0.03 ±0.001	302	0.03 ±0.001	128	0.334
	C	0.03 ±0.001	357	0.03 ±0.001	73	0.749
	D	0.03 ±0.001	337	0.03 ±0.001	93	0.638
C22:1 *trans*-13	*A*	0.07 ±0.002	53	0.07 ±0.002	377	0.706
	B	0.07 ±0.002	302	0.07 ±0.002	128	0.393
	C	**0.07 ±0.002**	**357**	**0.07 ±0.002**	**73**	**0.004**
	D	0.07 ±0.002	337	0.07 ±0.002	93	0.450
C20:5 *cis*-5,8,11,14,17	*A*	0.09 ±0.002	53	0.09 ±0.002	377	0.166
	B	0.09 ±0.002	302	0.09 ±0.002	128	0.270
	C	0.09 ±0.002	357	0.09 ±0.002	73	0.131

	D	0.09 ± 0.002	337	0.09 ± 0.002	93	0.687
C22:5 *cis*-7,10,13,16,19	*A*	0.13 ± 0.004	53	0.12 ± 0.003	377	0.436
	B	0.12 ± 0.003	302	0.12 ± 0.004	128	0.712
	C	0.12 ± 0.003	357	0.12 ± 0.004	73	0.853
	D	0.12 ± 0.003	337	0.13 ± 0.004	93	0.296
C24:0	*A*	0.05 ± 0.002	53	0.04 ± 0.001	377	0.240
	B	*0.04 ± 0.001*	*302*	*0.05 ± 0.001*	*128*	*0.060*
	C	0.04 ± 0.001	357	0.05 ± 0.002	73	0.507
	D	0.04 ± 0.001	337	0.05 ± 0.001	93	0.331
CLA *cis*-9, *trans*-11	*A*	0.94 ± 0.051	53	0.99 ± 0.040	377	0.265
	B	0.99 ± 0.041	302	0.95 ± 0.043	128	0.312
	C	0.97 ± 0.040	357	0.98 ± 0.049	73	0.747
	D	0.98 ± 0.040	337	0.96 ± 0.047	93	0.549

[1]The predicted means and standard error of those means were derived from the GLMMs. Cow age (age of the cow in years), herd and days in milk (DIM; being the integer number of days from the commencement of lactation for each individual cow), were fitted to the models as fixed effects.

[2]$P < 0.05$ in bold; $0.05 \leq P < 0.10$ in italics.

Supplementary Table S3. Associations between *MSTN* variant presence/absence and various grouped milk fatty acid (FA) component levels

Fatty acid (FA)[1]	Variant	Mean FA level ± SE[2] (g/100 g milk FA)				*P*[3]
		Absent	n	Present	n	
SCFA	A	4.00 ± 0.048	53	4.02 ± 0.037	377	0.531
	B	4.03 ± 0.037	302	4.00 ± 0.040	128	0.252
	C	4.02 ± 0.036	357	4.01 ± 0.045	73	0.871
	D	4.02 ± 0.036	337	4.00 ± 0.043	93	0.400
MCFA	*A*	19.64 ± 0.274	53	19.69 ± 0.216	377	0.841
	B	19.73 ± 0.218	302	19.59 ± 0.233	128	0.397
	C	19.63 ± 0.215	357	19.81 ± 0.261	73	0.397
	D	**19.78 ± 0.212**	**337**	**19.35 ± 0.251**	**93**	**0.021**
LCFA	*A*	48.26 ± 0.458	53	48.28 ± 0.361	377	0.962
	B	48.13 ± 0.364	302	48.51 ± 0.389	128	0.182
	C	48.39 ± 0.359	357	47.94 ± 0.436	73	0.207
	D	48.20 ± 0.357	337	48.52 ± 0.421	93	0.308
Omega 3	*A*	1.01 ± 0.020	53	0.99 ± 0.016	377	0.125
	B	1.00 ± 0.016	302	0.98 ± 0.017	128	0.107
	C	0.99 ± 0.016	357	1.01 ± 0.019	73	0.124
	D	0.99 ± 0.016	337	1.00 ± 0.019	93	0.345
Omega 6	*A*	0.847 ± 0.015	53	0.83 ± 0.012	377	0.141
	B	0.838 ± 0.012	302	0.82 ± 0.013	128	0.140
	C	0.829 ± 0.012	357	0.84 ± 0.015	73	0.245
	D	0.829 ± 0.012	337	0.84 ± 0.014	93	0.247
MUFA	A	20.24 ± 0.317	53	20.05 ± 0.243	377	0.499
	B	20.13 ± 0.248	302	20.04 ± 0.264	128	0.636
	C	20.06 ± 0.243	357	20.20 ± 0.301	73	0.562
	D	20.04 ± 0.242	337	20.24 ± 0.287	93	0.379
PUFA	*A*	3.94 ± 0.082	53	3.96 ± 0.065	377	0.776
	B	*3.99 ± 0.065*	*302*	*3.90 ± 0.070*	*128*	*0.094*
	C	3.94 ± 0.064	357	4.02 ± 0.078	73	0.193
	D	3.96 ± 0.064	337	3.95 ± 0.076	93	0.891
branched FA	*A*	1.58 ± 0.026	53	1.59 ± 0.020	377	0.558
	B	1.60 ± 0.021	302	1.58 ± 0.022	128	0.155
	C	1.58 ± 0.020	357	1.61 ± 0.025	73	0.209
	D	1.60 ± 0.020	337	1.57 ± 0.024	93	0.196
C12:1 index	*A*	2.23 ± 0.062	53	2.29 ± 0.049	377	0.276
	B	2.30 ± 0.049	302	2.24 ± 0.053	128	0.104
	C	2.28 ± 0.049	357	2.26 ± 0.059	73	0.686
	D	2.28 ± 0.048	337	2.26 ± 0.057	93	0.573

C16:1 index	*A*	3.52 ±0.097	53	3.50 ±0.077	377	0.831
	B	3.53 ±0.078	302	3.46 ±0.083	128	0.243
	C	3.50 ±0.076	357	3.52 ±0.093	73	0.709
	D	3.47 ±0.076	337	3.59 ±0.089	93	0.083
C18:1 index	A	65.77 ±0.603	53	66.01 ±0.463	377	0.643
	B	66.08 ±0.472	302	65.78 ±0.502	128	0.438
	C	65.97 ±0.461	357	65.93 ±0.572	73	0.942
	D	65.90 ±0.460	337	66.13 ±0.546	93	0.589

[1]SCFA = Short Chain Fatty Acid, MCFA = Medium Chain Fatty Acid, LCFA = Long Chain Fatty Acid, PUFA = Polyunsaturated Fatty Acid, MUFA = Monounsaturated Fatty Acid.

[2]The predicted means and standard error of those means were derived from the GLMMs. Cow age (age of the cow in years), herd and days in milk (DIM; being the integer number of days from the commencement of lactation for each individual cow) were fitted to the models as fixed effects.

[3]$P < 0.05$ in bold; $0.05 \leq P < 0.10$ in italics.
